# Tumour-agnostic immunotherapy: aligning biological rationale, biomarker precision, and real-world implementation

**DOI:** 10.1016/j.eclinm.2026.104211

**Published:** 2026-09-23

**Authors:** Jia Liu, Juliana Beal, Niamh Coleman, James Lynam, Vivek Subbiah

**Affiliations:** a The Kinghorn Cancer Centre, St Vincent's Hospital, Sydney, NSW, Australia; b School of Clinical Medicine, Faculty of Medicine & Health, University of New South Wales, Sydney, Australia; c Garvan Institute of Medical Research, Sydney, NSW, Australia; d ProCan®, Children’s Medical Research Institute, Faculty of Medicine & Health, University of Sydney, Westmead, NSW, Australia; e Hospital Israelita Albert Einstein, Sao Paulo, Brazil; f Trinity St. James's Cancer Institute, James Street, Dublin 8, Ireland; g Medical Oncology Department, St. James's Hospital, Dublin 8, Ireland; h School of Medicine, Trinity College Dublin, Ireland; i Cancer Institute NSW, Australia; j School of Medicine and Public Health, University of Newcastle, NSW, Australia; k Stanford Cancer Institute, Stanford, CA, USA

**Keywords:** Tumour-agnostic therapy, Immune checkpoint inhibitors, Precision oncology, Rare cancers, Health-system implementation

## Abstract

Tumour-agnostic immunotherapy has reshaped precision oncology by showing that treatment benefit can, in selected settings, be predicted by a shared biological state rather than anatomical origin. Microsatellite instability-high or mismatch repair-deficient cancer is the clearest example, with durable responses across diverse malignancies. By contrast, broader biomarkers show that pan-cancer applicability cannot be inferred from a measurable molecular feature alone. Tumour lineage, antigen presentation, immune microenvironment, prior therapy, and health-system context all influence whether a tumour-agnostic indication translates into real-world benefit. We examine the biological principles that distinguish robust from context-dependent immunotherapy biomarkers, the uncertainty created by rare cancers and incomplete evidence, and the implementation challenges that arise when therapeutic access outpaces biomarker infrastructure. Using Australia's broad reimbursement of immune checkpoint inhibitors for immunotherapy-sensitive advanced cancers as a case study, we consider implications for emerging pan-tumour platforms. We argue for biologically informed access embedded within learning health systems.

**Search strategy and selection criteria**

This was a targeted narrative review rather than a systematic review. We searched PubMed/MEDLINE, Embase, ClinicalTrials.gov, regulatory agency documents, Pharmaceutical Benefits Scheme and Pharmaceutical Benefits Advisory Committee documents, and relevant oncology conference proceedings for articles published up to 18th August 2026. Search terms included “tumour-agnostic”, “tissue-agnostic”, “immunotherapy”, “checkpoint inhibitor”, “MSI-H”, “dMMR”, “TMB”, “POLE”, “POLD1”, “PD-L1”, “tumor microenvironment”, “biomarker”, “rare cancer”, “basket trial”, “nivolumab”, “ipilimumab”, “pembrolizumab”, “dostarlimab”, “reimbursement”, “precision oncology”, and “implementation”.

We prioritised prospective clinical trials, regulatory approvals, health-technology assessment documents, major reviews, and real-world evidence relevant to tumour-agnostic immunotherapy indications or reimbursement; studies evaluating the biological or clinical validity of candidate immunotherapy biomarkers; and real-world or implementation studies relevant to patient selection and delivery of tumour-agnostic immunotherapy. Landmark mechanistic studies and major reviews were included where they informed biological interpretation or contextualised clinical evidence. Small retrospective series and conference reports were considered when prospective evidence was limited, particularly for rare cancers. Case reports were not routinely included. References were selected for relevance to biological rationale, clinical evidence, access, reimbursement, toxicity, and implementation.

## Introduction

The organ of origin has defined cancer disease classification, shaped research agendas, and guided treatment selection for over a century. However, this histological framework obscures the fact that tumours arising in vastly different tissues can harbour similar molecular liabilities. The watershed moment came in 2017, when the US Food and Drug Administration approved pembrolizumab for patients with microsatellite instability-high (MSI-H) or mismatch repair-deficient (dMMR) cancers, irrespective of tissue type.^1^, ^2^, ^3^ This unprecedented decision marked the emergence of tissue-agnostic oncology: a recognition that the molecular signature of a cancer, not the organ harbouring it, could dictate treatment selection.^4^ Since that landmark approval, a growing number of genomic features spanning gene fusions/rearrangements (*NTRK, RET*), mutations (*BRAF V600E*), high tumour mutational burden (TMB-H), HER2 overexpression (HER2 IHC 3+) have crossed traditional cancer boundaries to define new therapeutic classes.^5^ This shift carries significant implications for drug development, clinical trial design, and equitable access to precision therapies across diverse cancer types united by shared biomarkers.

However, approved tumour-agnostic biomarkers remain limited: while MSI-H/dMMR has demonstrated durable activity across malignancies, TMB-H has yielded inconsistent clinical results.^6^^,^^7^ Other tissue-agnostic immune sensitive features, such as *POLE* mutations^8^ and *PD-L1* gene amplification,^9^ lack widespread testing availability and regulatory approval. The central challenge is identifying biomarkers that capture biologically meaningful vulnerabilities, not merely those that occur across multiple tumour types. Clinicians increasingly encounter patients with rare cancers, exhausted standard options, and biomarkers that are suggestive but unproven. In these settings, the decision to treat sits between biological rationale, incomplete evidence, patient preference, and reimbursement constraints.^10^ Tumour-agnostic immunotherapy thus raises critical questions: What makes a biomarker sufficiently valid? When does tumour lineage still matter? How should clinicians act with incomplete evidence? What infrastructure ensures equitable access rather than ‘try it and see’ and low-value care?

These questions are becoming more urgent as health systems begin to fund immunotherapy beyond conventional tumour-specific indications. Australia illustrates this challenge as a case study. In 2026, national public reimbursement for ipilimumab plus nivolumab and pembrolizumab provides expanded access for ‘all immune-sensitive advanced or metastatic tumours’ including rare and uncommon cancers lacking conventional trial data.^11^, ^12^, ^13^ This broadens equity but exposes a critical gap: drugs are funded more broadly than the evidence base, biomarker testing, expert decision support and registry infrastructure needed to deploy them safely in the clinic.^14^

In this perspective, we examine implementation challenges in tumour-agnostic immunotherapy across biology, biomarker precision, clinical judgment, and health-system practice. While biomarker-driven approaches are scientifically rigorous, they may leave many patients, particularly those with rare cancers lacking data, without access to testing or treatment options. Conversely, broadening access without biomarkers demands refined clinical judgment to optimize who, when, how, and why patients receive immunotherapy, given that the science of immune sensitivity remains incomplete. We contrast MSI-H/dMMR success with TMB's context dependency, consider how tumour lineage, microenvironment, and prior treatment modulate response, and address the clinical grey zone where immunotherapy sensitivity exists without validated biomarkers. Using Australia as a real-world case study, we extend these lessons to emerging modalities including ADCs, bispecific antibodies, and other emerging pan-tumour platforms.

## Biological states versus molecular surrogates

The most effective tumour-agnostic biomarkers identify conserved biological phenotypes, not indirect molecular features (Fig. 1A). MSI-H/dMMR exemplifies this distinction. Defective mismatch repair generates frameshift neoantigens that activate a characteristic immune state: robust CD8+ infiltration, heightened interferon signaling, and adaptive PD-1 upregulation.^1^^,^^15^^,^^16^ Critically, this immune phenotype is sufficiently conserved across multiple tumour types to support tumour-agnostic activity, although response magnitude remains heterogeneous across lineages and molecular contexts.^15^ In KEYNOTE-158, pembrolizumab achieved durable responses in roughly one-third of patients across >25 malignancies despite vast differences in tissue of origin, a triumph stemming not from a shared mutation, but from a shared immune sensitivity.^2^ The tumour-agnostic dMMR paradigm subsequently extended beyond pembrolizumab, with the US FDA granting accelerated approval to dostarlimab for previously treated recurrent or advanced dMMR solid tumours in 2021, supported by the GARNET study.^17^

**Fig. 1:**
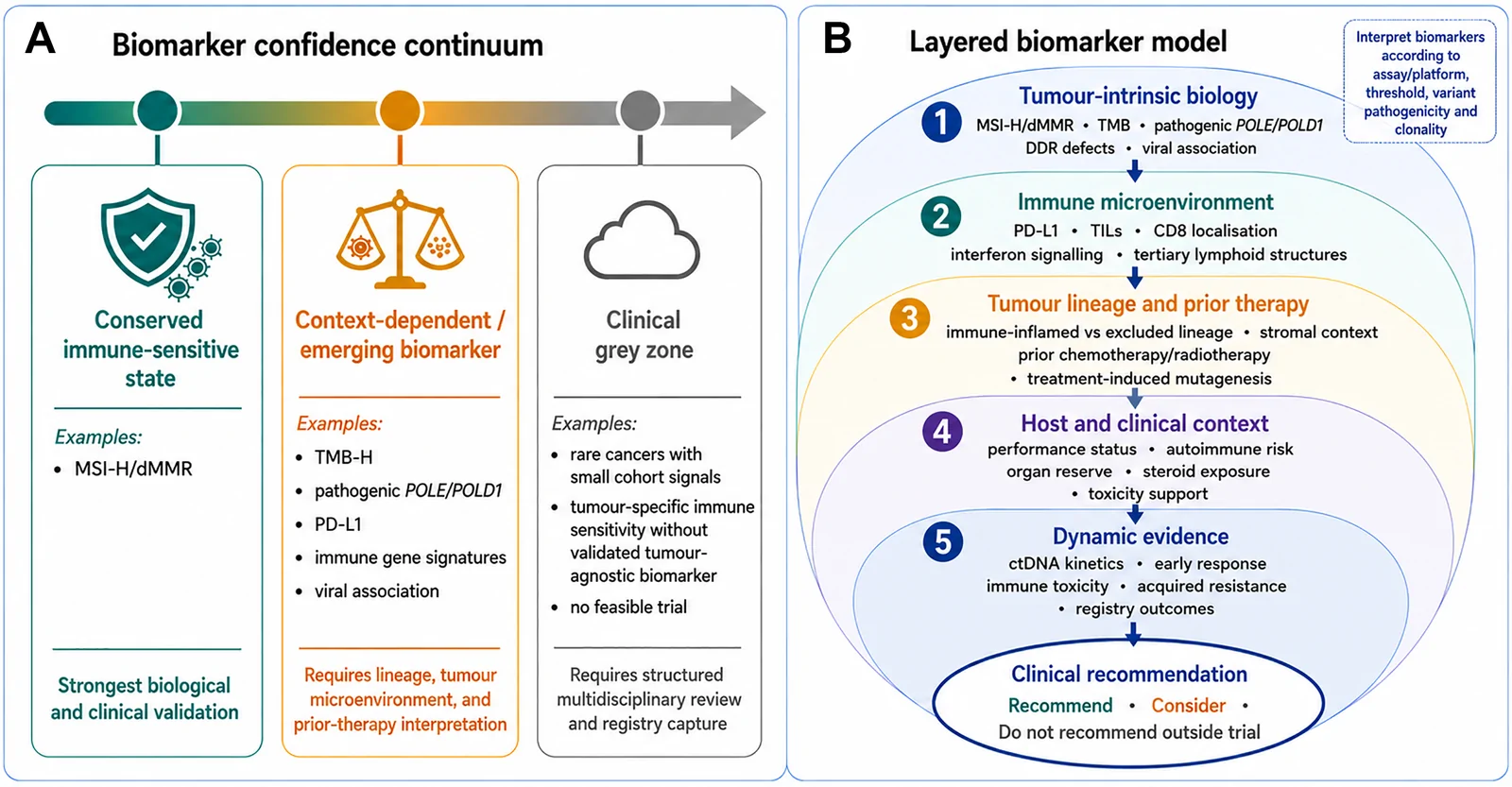
**A layered framework for tumour-agnostic immunotherapy decision making**. A: Tumour-agnostic immunotherapy biomarkers vary in biological confidence, from conserved immune-sensitive states such as MSI-H/dMMR, to context-dependent markers such as TMB (although this is a FDA-approved tumour-agnostic biomarker), PD-L1, and immune gene signatures, to rare-cancer scenarios where immune sensitivity may be plausible but incompletely captured by validated biomarkers. B: Clinical decision-making should integrate tumour-intrinsic biology, immune microenvironment, tumour lineage and prior therapy, host and clinical context, and dynamic evidence, including ctDNA kinetics, early response, immune toxicity, acquired resistance, and registry outcomes. Image created with assistance of ChatGTP Plus (OpenAI).

TMB reveals the converse principle. Although the premise was compelling, more mutations should yield more neoantigens and greater checkpoint-inhibitor sensitivity, the pan-cancer experience exposed a fundamental limitation.^18^^,^^19^ A TMB threshold of 10 mutations/Mb conflates biologically disparate cancers with different mutational processes, neoantigen quality, antigen presentation machinery, and immune microenvironments.^20^, ^21^, ^22^ TMB quantifies mutation burden while missing immunogenicity, T-cell infiltration, and immune exclusion, all determinants of clinical response. Accordingly, its predictive value varies substantially by tumour type, remaining strong only in cancers already characterized by immune infiltration and this is reflected in real-world datasets showing variable efficacy of pembrolizumab in different solid tumours that are TMB-H.^23^ The immune ecosystem of a pancreatic adenocarcinoma, for instance, differs fundamentally from that of a melanoma or endometrial carcinoma, regardless of whether a shared biomarker is present.^21^^,^^22^ There are also increasing data to suggest that the tumour microenvironment, rather than the molecular alteration itself, may ultimately determine whether an immune-sensitive state can be established and maintained.^21^^,^^22^

Nevertheless, the US FDA's 2020 accelerated approval of pembrolizumab for previously treated unresectable or metastatic TMB-high solid tumours, defined as ≥10 mutations/Mb by an FDA-approved test, provided a tumour-agnostic treatment option for selected patients without satisfactory alternatives.^19^ The challenge lies in learning from imperfect biomarkers while systematically improving their precision. The principle remains: tissue-agnostic biomarkers succeed by identifying conserved biological mechanisms, but implementation must balance scientific rigor with equitable access.^10^

## Immunotherapy biomarkers: from single biomarkers to layered decision-making

The experience of tumour-agnostic immunotherapy shows that biomarkers should not be viewed as simply tissue-specific or tissue-independent. Most clinically useful biomarkers sit between these poles. MSI-H/dMMR comes closest to identifying a conserved immune-sensitive state across tumour types. TMB illustrates the opposite problem: although it can be measured across cancers, its clinical meaning depends heavily on the mutational process that generated it, the quality of neoantigens, immune infiltration, antigen presentation, and whether the tumour lineage is permissive to immune recognition.^23^

A more useful framework is to consider immunotherapy biomarkers as layered rather than binary. Tumour-intrinsic markers include MSI-H/dMMR, TMB, *POLE/POLD1* mutations, viral antigens, DNA damage repair defects, mutational signatures, HLA loss, and oncogenic pathways associated with immune exclusion.^15^^,^^22^ Immune microenvironment markers include PD-L1 expression, tumour-infiltrating lymphocytes, CD8+ T-cell localisation, interferon signaling, myeloid infiltration, tertiary lymphoid structures, and spatial immune organization.^16^^,^^21^^,^^24^ Host and systemic markers include immune fitness, microbiome composition, corticosteroid exposure, autoimmune risk, organ reserve, and comorbidities that influence both benefit and toxicity. Dynamic markers include ctDNA kinetics, early radiographic patterns, immune-related toxicity, and on-treatment changes in immune activation or resistance^25^^,^^26^ (Fig. 1B).

This layered model helps explain why single biomarkers often perform inconsistently when moved into tumour-agnostic practice. A high TMB result may be less informative when it reflects treatment-induced hypermutation such as temozolomide-associated, mismatch-repair-deficient hypermutation in recurrent glioma rather than a clonal endogenous immune-sensitive state.^27^

These candidate biomarkers differ substantially in analytical and clinical validation. PD-L1 expression has established tumour-specific assays and clinical utility, but its predictive value depends on assay, scoring method, threshold, and tumour lineage^28^; PD-L1 amplification has considerably less clinical validation and no established pan-cancer selection standard.^9^ Similarly, the relevance of POLE/POLD1 alterations depends on variant pathogenicity and genomic context,^29^ while viral association may identify immune-sensitive biology in selected virus-associated malignancies but is not a validated tumour-agnostic biomarker.^30^ Table 1 provides a structured evidence table for existing immunotherapy-relevant biomarkers.

**Table 1:** Evidence supporting biomarkers and biological states associated with immune checkpoint inhibitor sensitivity across solid tumours.

Biomarker or biological state	Biological rationale	Assay and clinically relevant threshold	Key evidence	Regulatory status	Important lineage, assay and context specifications	Level of clinical actionability
MSI-H/dMMR	Loss of mismatch repair generates insertion/deletion mutations and frameshift neoantigens, producing an immune-inflamed phenotype.	dMMR: loss of MLH1, PMS2, MSH2 and/or MSH6 by validated IHC. MSI-H: validated PCR or NGS-based MSI assay. No universal MSI threshold across platforms.	KEYNOTE-158, ORR 33.8%, with median duration of response 63.2 months.^2^ Phase 1 GARNET trial, ORR 44%, duration response 27.7 months^17^	Validated tumour-agnostic biomarker. US FDA tissue-agnostic pembrolizumab indication established in 2017 and dostarlimab in 2021.	Not completely lineage-independent. Response probability and magnitude vary with tumour lineage, underlying mechanism of MMR deficiency, indel/neoantigen burden and immune context.	**Highest actionability: validated tumour-agnostic state.** Strongly supports treatment when the treatment setting, alternatives and patient-specific benefit–risk are appropriate.
TMB-High	Greater somatic mutation burden increases probability of neoantigen generation that is recognisable by T cells, providing the rationale for immunotherapy sensitivity. Does not account for quality of antigen, presentation or tumour microenvironment.	Tissue TMB using a validated NGS platform: ≥10 mutations/Mb by an FDA-approved test; FoundationOne CDx was approved as a companion diagnostic. TMB values are not directly interchangeable across panels without appropriate calibration.	KEYNOTE-158, 102 patients with tissue TMB-H tumours across 10 specific histologies with ORR 29%.^18^ Real world evidence demonstrates significant tumour-type heterogeneity.	US FDA accelerated tumour agnostic approval of pembrolizumab for TMB-high (≥10 mut/Mb) solid tumours without satisfactory alternatives (2020).	Strong assay dependence; panel size, variant filtering and bioinformatics affect TMB. Baseline TMB distributions differ between lineages. Mutational process, clonality, immune infiltration and antigen-presentation competence matter.	**Regulatory but context-dependent.** Should strengthen rather than determine a treatment recommendation.
Pathogenic POLE/POLD1 proofreading deficiency	Functionally deleterious polymerase proofreading defects produce an ultramutated phenotype, high neoantigen burden and enhanced tumour immunogenicity.	Tumour NGS with variant-level pathogenicity assessment. Interpretation should distinguish pathogenic proofreading/exonuclease-domain alterations from VUS/passenger mutations. No validated pan-cancer threshold.	Retrospective analyses correlate pathogenic POLE/POLD1 mutations with higher mutations/megabase and checkpoint inhibitor efficacy.^8^	No established tumour-agnostic regulatory indication based solely on POLE/POLD1 status.	Variant location/function, mutational signature, TMB, MSI status and clonality matter. Clinical evidence is strongest in CRC and endometrial cancer and remains considerably less mature in other lineages.	**Strong emerging/context-dependent biomarker.** Can provide compelling biological support when a bona fide proofreading-deficient phenotype is demonstrated.
PD-L1 protein expression	PD-L1 expression may reflect adaptive immune resistance in an antigen-experienced, interferon-activated microenvironment and identifies ICI-responsive populations in several tumour types.	IHC using the drug- and tumour-specific validated assay and scoring algorithm. Assays use TPS, CPS or immune-cell scores and different thresholds. No validated pan-cancer threshold.	Prospectively evaluated as a histology–drug pair. Higher expression generally translates to better responses, however there are still outliers.	Multiple tumour-specific regulatory indications/CDx assays; no PD-L1-expression-defined tumour-agnostic ICI indication.	Assay clone, platform, scoring method and cut-off; interobserver variability; tumour versus immune-cell expression; spatial and temporal heterogeneity all modify interpretation.	**Clinically actionable in validated tumour-specific contexts**; otherwise supportive/contextual. Not a universal pan-cancer biomarker.
CD274/PD-L1 gene amplification	Increased CD274 gene dosage may drive PD-L1 overexpression and immune evasion, providing a mechanistic rationale for PD-1/PD-L1 blockade.	In situ hybridization of CNV by NGS. No validated threshold. Amplification does not linearly correlate to IHC expression.	In a pan-cancer series, CD274/PD-L1 amplification was rare (0.7%) but distributed across >100 histologies.^9^ Nationwide CGP-based retrospective study found greater ICI activity in CD274-amplified solid tumours, supporting further validation but not establishing pan-cancer selection standard.^31^	No tumour-agnostic regulatory indication or approved pan-cancer CDx.	Rare alteration; definition of amplification varies by sequencing platform and tumour purity. Gene amplification and PD-L1 protein expression are related but not equivalent; co-amplified 9p24 genes and tumour lineage may influence phenotype.	**Emerging/supportive biomarker**. Appropriate as an additional biological signal, not a standalone indication for treatment outside an established tumour-specific context or trial.
T-cell inflamed/interferon-γ gene-expression signatures	Expression of interferon-responsive genes, antigen-presentation machinery, chemokines and T-cell markers reflects a pre-existing antitumour immune response that may be reinvigorated by checkpoint blockade.	RNA-based multigene expression platforms; individual signatures and cut-offs differ. No harmonised pan-cancer clinical assay or universal threshold.	T-cell-inflamed gene expression profile and TMB provided complementary predictive information across pembrolizumab-treated tumour cohorts, supporting layered rather than single-biomarker assessment.^32^	No established pan-cancer regulatory CDx.	Platform/signature composition, tissue quality, stromal and immune-cell admixture and pre-treatment sampling affect results.	**Emerging supportive biomarker.** Useful conceptually and potentially for clinical trials; insufficient for routine standalone prescribing.
Virus-associated immune biology	Viral antigens can provide highly foreign tumour antigens and induce T-cell activation, interferon signalling and adaptive PD-L1 expression.	Virus- and lineage-specific assays: eg, EBER in-situ hybridisation for EBV; HPV DNA/RNA testing with p16 as a context-specific surrogate; MCPyV assays in Merkel cell carcinoma. No universal viral assay or threshold.	In a prospective phase 2 metastatic gastric cancer study, EBV-positive tumours showed striking pembrolizumab activity, but the cohort was small.^30^ Robust ICI activity also occurs in Merkel cell carcinoma, although response is not restricted to virus-positive disease.	No regulatory tumour-agnostic indication based on viral positivity itself. Several virus-associated cancers have tumour-specific ICI indications.	The biological consequences of oncogenic viruses differ substantially. Viral positivity may be prognostic, predictive, or simply associated with a particular immune phenotype depending on lineage.	**Tumour-specific/context-dependent supportive biomarker.** Should not be described as a validated tumour-agnostic biomarker.
Immune-infiltrated/spatial phenotype: TILs, CD8 localisation and tertiary lymphoid structures (TLS)	Pre-existing effector-cell infiltration and organised local antigen presentation indicate an immune-permissive microenvironment; spatial exclusion of effector cells may indicate resistance despite tumour-intrinsic immunogenicity.	H&E/IHC, multiplex immunofluorescence, digital pathology or spatial profiling. Methods for TIL density, CD8 localisation and TLS presence/maturity differ; no universal validated threshold.	Multiple tumour-specific studies associate TILs/TLS with ICI response. In a multi-tumour analysis, mature TLS were associated with improved response, PFS and OS independently of PD-L1 expression and CD8 density.^33^	No established pan-cancer regulatory CDx	Highly spatial and sampling-dependent; maturity and location of TLS may matter. Associations may be partly prognostic rather than purely predictive.	**Emerging supportive biomarker.** Particularly valuable as a second layer of evidence when genomic biomarkers are equivocal; not yet a routine standalone treatment criterion.
Antigen-presentation and immune-exclusion/resistance alterations (eg, B2M/HLA loss, JAK1/2 loss; lineage-specific STK11/KEAP1 or WNT/β-catenin biology)	Loss of antigen presentation, interferon responsiveness or T-cell trafficking can uncouple tumour mutation/neoantigen generation from effective immune recognition.	NGS, copy-number analysis and/or IHC depending on alteration. No validated pan-cancer negative threshold.	Mechanistic and clinical studies support B2M/JAK pathway alterations as resistance mechanisms; STK11/LKB1 is associated with resistance to PD-1-axis monotherapy particularly in KRAS-mutant lung adenocarcinoma.	No regulatory pan-cancer negative biomarker.	Effects are highly alteration-, treatment- and lineage-specific and are not absolute.	**Contextual negative modifier,** not an absolute contraindication. May lower confidence in otherwise favourable biomarkers and support trial/MDT review.

Abbreviations: B2M, beta-2-microglobulin; CDx, companion diagnostic; CGP, comprehensive genomic profiling; CNV, copy-number variation; CPS, combined positive score; CRC, colorectal cancer; dMMR, deficient mismatch repair; FDA, US Food and Drug Administration; H&E, haematoxylin and eosin; HLA, human leukocyte antigen; ICI, immune checkpoint inhibitor; IHC, immunohistochemistry; MSI-H, microsatellite instability-high; NGS, next-generation sequencing; ORR, objective response rate; OS, overall survival; PCR, polymerase chain reaction; PD-1, programmed cell death protein 1; PD-L1, programmed death-ligand 1; PFS, progression-free survival; TIL, tumour-infiltrating lymphocyte; TLS, tertiary lymphoid structure; TMB, tumour mutational burden; TMB-H, tumour mutational burden-high; TPS, tumour proportion score; VUS, variant of uncertain significance.

Biomarkers are ordered broadly according to biological and clinical validation rather than as an additive ranking or scoring system. Clinical actionability reflects the authors' synthesis of current evidence and should be interpreted alongside tumour lineage, strength of tumour-specific clinical evidence, assay characteristics and threshold, variant pathogenicity and clonality, treatment line, prior therapy, competing treatment options, and patient-specific benefit–risk. A tumour-agnostic biomarker denotes evidence or regulatory recognition across multiple histologies and does not imply uniform response probability or magnitude of benefit across tumour types. Regulatory status refers to US Food and Drug Administration (FDA) indications unless otherwise specified; regulatory approvals, companion diagnostic requirements, and reimbursement vary between jurisdictions.

The practical challenge is therefore not only biomarker discovery, but real world implementation and access to informative biomarkers to assist with patient selection for immunotherapy. A biomarker must be analytically reliable, biologically interpretable, clinically actionable, affordable, and available quickly enough to guide treatment.^34^ Tumour-agnostic immunotherapy requires a tiered approach: first, identify established immune-sensitive states such as MSI-H/dMMR; second, interpret broader markers such as TMB, PD-L1, POLE/POLD1, viral association, and immune infiltration within tumour-lineage and clinical context; third, use structured multidisciplinary review, including rare cancer or molecular tumour boards where available, together with prospective registries, to support decisions where evidence is incomplete. This approach preserves the promise of tumour-agnostic access while avoiding the misconception that any measurable pan-cancer feature is automatically a pan-cancer predictor of benefit.

## Rare cancers and the clinical grey zone

Rare tumours expose a fundamental limitation of the tumour-agnostic paradigm: many are systematically excluded from pivotal trials and yet demonstrate immunotherapy sensitivity without validated predictive biomarkers (Fig. 1A). Examples include virus-associated tumours such as Merkel cell carcinoma, as well as alveolar soft-part sarcoma, thymic carcinoma, and selected sarcoma or neuroendocrine subtypes.^33^^,^^35^^,^^36^ Importantly, these examples represent tumour-specific evidence of immune sensitivity rather than validated tumour-agnostic biomarkers. Their relevance to tumour-agnostic implementation lies in illustrating the clinical grey zone in which immunotherapy activity may be established within a rare tumour type despite the absence of a biomarker capable of predicting benefit across histologies.

Basket and platform trials in rare cancers have documented meaningful responses in patient subsets, yet predictive biomarkers remain incompletely defined. The SWOG S1609 DART trial (NCT02834013) was a prospective, multicentre, open-label phase 2 basket trial of ipilimumab plus nivolumab in rare cancers, opened across more than 1000 US National Clinical Trials Network sites. Across 53 rare cancer cohorts, 798 patients were enrolled and 727 eligible patients received treatment; 24 of 53 cohorts showed clinical activity, with an overall confirmed response rate of 13%.^37^ Durable responses were observed in several rare cancer cohorts, including angiosarcoma, non-pancreatic neuroendocrine tumours, high-grade neuroendocrine neoplasms, and rare gynecological malignancies, although correlative studies to define response and resistance biomarkers remain ongoing.^37^, ^38^, ^39^, ^40^, ^41^ The Australian and New Zealand MoST-CIRCUIT trial provides a complementary rare-cancer example within a precision oncology infrastructure. This prospective, multicentre phase II trial evaluated nivolumab plus ipilimumab in selected immunotherapy-sensitive advanced rare cancers across 17 sites, including neuroendocrine, biliary tract, gynecological, and non-colorectal dMMR/MSI-H cancers. Published cohort results showed clinically meaningful activity, including an objective response rate of 54% in advanced ovarian and endometrial clear cell cancers, despite most tumours being mismatch repair-proficient, microsatellite-stable, and low TMB,^42^ and an objective response rate of 63% in non-colorectal dMMR/MSI-H cancers across 17 tumour types.^43^ Together with DART, MoST-CIRCUIT illustrates that rare-cancer basket trials can reveal immunotherapy-sensitive tumour cohorts, while prospective biomarkers capable of identifying individual responders remain limited.

This creates a clinical dilemma: these patients often have exhausted conventional therapies, and the biological rationale for immunotherapy sensitivity may be compelling, but clinicians lack precise biomarkers in rare cancer settings to identify who will benefit. Clinicians are therefore left to make decisions using incomplete data: biological plausibility, small cohort signals, extrapolation from related tumour types, patient preference, toxicity risk, and availability of trials or reimbursed treatment.

This is the clinical grey zone of tumour-agnostic immunotherapy. A patient may present with a rare cancer, limited standard options, emerging evidence of immunotherapy activity, no validated biomarker, no reimbursed molecular test, and no feasible trial. In this setting, a strictly biomarker-defined access model may be too restrictive, but unrestricted prescribing risks unwarranted toxicity, false hope, and low-value care. The central question is therefore not whether all rare cancers should be treated as tumour-agnostic, but how clinicians and health systems should make transparent, evidence-informed decisions when certainty is impossible.

Rare cancer networks, molecular and rare tumour boards, prospective registries, and structured treatment rationales are essential infrastructure in this setting. They do not eliminate uncertainty, but they make uncertainty explicit, support consistency in decision-making, and convert individual clinical judgement into collective learning. Rather than viewing rare cancers as outliers, tumour-agnostic immunotherapy should treat them as a test case for whether precision oncology can generate evidence for patients who would otherwise remain invisible in conventional clinical trial datasets.

## Australia as an implementation case study

Australia offers an instructive case in the opportunities and implementation barriers posed by tumour-agnostic immunotherapy reimbursement policy. The Australian system separates regulatory approval from public reimbursement. The Therapeutic Goods Administration approval determines whether a medicine can legally be supplied, but universal public subsidy through the Pharmaceutical Benefits Scheme requires a separate Pharmaceutical Benefits Advisory Committee (PBAC) recommendation to the government and funding approval, based on comparative clinical benefit, safety, cost-effectiveness, and budget impact.^44^ Once listed, the Pharmaceutical Benefits Scheme supports nationally consistent access with capped patient co-payments, largely independent of private insurance coverage.

Diagnostic tests and medical services are assessed and funded through a separate Medical Services Advisory Committee process and funded through the Medicare Benefits Schedule.^45^ This separation creates a distinctive challenge for precision oncology: a medicine may be approved but not reimbursed, a biomarker test may be scientifically important but not publicly funded, and the therapeutic and diagnostic pathways may not be assessed or implemented in parallel. This matters acutely for tumour-agnostic immunotherapy access. The clinical rationale for pan-cancer prescribing depends on the presence of a shared immune-sensitive biology, but the health-system pathway needed to deliver treatment is not biology agnostic.^14^ Prior to broad reimbursement, patients with rare cancers often depended on tumour-specific listings, compassionate access, private payment, or exceptional pathways.

Australia's 2026 PBS changes consolidated and expanded reimbursement of ipilimumab plus nivolumab and pembrolizumab for eligible patients with advanced or metastatic cancers considered “immunotherapy-sensitive”.^11^, ^12^, ^13^ This should not be interpreted as unrestricted reimbursement for any malignancy. Importantly, the PBS reimbursement changes did not create a corresponding tumour-agnostic TGA indication for these agents. Reimbursement eligibility, regulatory approval, and the clinical judgement that immunotherapy is appropriate for an individual patient therefore remain distinct considerations. Within the reimbursement criteria, clinicians must still determine whether treatment is supported by the available clinical evidence, tumour biology, competing treatment options, and patient-specific benefit–risk. The breadth of the reimbursement model nevertheless places greater responsibility on clinicians to determine what constitutes sufficient evidence of “immunotherapy sensitivity” for appropriate clinical use, particularly for rare cancers where prospective data and validated predictive biomarkers may be limited.

This world first funding approach, the result of significant partnership between industry, government, clinical and patient advocate bodies, offers several important advantages. It creates national access to immune checkpoint inhibitors for patients with rare and uncommon cancers, including those historically excluded from tumour-specific pivotal trials. It also allows clinicians to consider immunotherapy across a wider range of tumour types when conventional treatment options are exhausted or poorly supported by evidence. It also bypassed the significant time delays and financial implications faced due to the prior requirement of individual tumour specific submissions. Importantly, the model includes shared cost between government and sponsor and creates the possibility of a learning health system, with reassessment after three years.^14^ For patients and clinicians, this represents a major shift from exceptional access or compassionate-use pathways toward a national reimbursement mechanism for pan-cancer immunotherapy with improved equity of access particularly for patients with rare cancers.

However, the same decision also exposes the implementation risks of access that is broader than the underlying evidence base.^46^ The listing does not require a positive biomarker, and broad pan-cancer next-generation sequencing is not currently universally funded. This means Australia may be one of the first countries to fund pan-cancer immunotherapy without simultaneously funding the biomarker testing infrastructure.^46^ In practice, this transfers substantial responsibility to the treating clinician.^47^ Clinicians must decide whether a cancer is sufficiently “immunotherapy sensitive,” whether available evidence is strong enough in a rare tumour type, whether a biomarker result is necessary or helpful.

## A pragmatic decision framework

The central implementation question is therefore how “immunotherapy-sensitive” should be defined for treatment and reimbursement. We propose that ‘immunotherapy-sensitive’ should not be determined by tumour type or a single biomarker alone, but through four linked domains: strength of clinical evidence, biological/biomarker evidence within tumour-lineage context, competing treatment options, and patient-specific benefit–risk (Table 2). Evidence should be weighted hierarchically: prospective clinical data and validated immune-sensitive states provide stronger support than small basket cohorts, retrospective evidence, or context-dependent biomarkers. Importantly, broad reimbursement should not displace more evidence-based standard therapy or feasible clinical trial participation, and the potential for meaningful benefit must be balanced against individual toxicity risk.

**Table 2:** Pragmatic decision framework for pan-cancer immunotherapy prescribing.

Domain	Supports treatment	Context-dependent/uncertain	Argues against treatment
Clinical evidence of ICI sensitivity	Prospective Phase II-III trials demonstrating meaningful activity in the tumour type or relevant biological subgroup	Small prospective cohorts/basket trials; consistent retrospective or real-world evidence	Negative prospective evidence or absence of meaningful supporting evidence
Biological/biomarker evidence	Validated immune-sensitive state, e.g. MSI-H/dMMR; other biomarkers with strong evidence in the relevant lineage/context.	PD-L1/TMB-H but uncertain evidence, virally induced, immune-inflamed phenotype where predictive relevance is context-dependent	Biology associated with ICI resistance in the relevant tumour context; biomarker of uncertain pathogenicity/assay validity
Competing treatment options	No superior evidence-based standard therapy or suitable clinical trial	Alternative options exist but expected benefit is limited/uncertain	Clearly superior evidence-based therapy or appropriate biomarker-directed treatment available
Patient-specific benefit-risk	Performance status, organ reserve and comorbidity profile support treatment; toxicity can be appropriately managed	Increased toxicity risk requiring individualised assessment	Risk of serious toxicity likely to outweigh plausible benefit

Recommendation	Suggested interpretation
Recommend	Convincing clinical evidence and/or a validated immune-sensitive biological state in the relevant context, no clearly superior treatment option, and an acceptable patient-specific benefit–risk profile
Consider with multidisciplinary review	Some evidence or biological rationale for ICI sensitivity, but uncertainty remains regarding magnitude of benefit, biomarker validity, tumour lineage or individual patient factors. Multidisciplinary review and prospective outcome capture should be considered
Do not recommend outside a clinical trial	Little or negative evidence of ICI sensitivity without compelling biological rationale; a clearly superior evidence-based alternative exists; or treatment-related risk is judged to outweigh the plausible benefit

These domains are not intended as an additive numerical score. Their relative weight varies by tumour lineage and clinical context. Strong positive evidence (for example, a validated immune-sensitive state) may outweigh uncertainty in other domains, whereas major safety concerns or a clearly superior standard therapy may override otherwise favourable features. The significance of an individual biomarker depends on tumour lineage, assay characteristics, variant pathogenicity and clonality, treatment line, and prior therapy.

These domains should then be integrated into an overall recommendation to 1) recommend, 2) consider with multidisciplinary review or 3) not recommend immunotherapy outside a clinical trial. Treatment can be recommended when convincing clinical evidence and/or a validated immune-sensitive biological state is present in the relevant context, no clearly superior treatment option exists, and the anticipated benefit–risk profile is acceptable. Immunotherapy may be considered where evidence or biological rationale suggests potential sensitivity but important uncertainty remains regarding the magnitude of benefit, biomarker validity, tumour lineage, or individual patient factors; structured multidisciplinary review and prospective outcome capture are particularly valuable in this setting. Conversely, treatment should generally not be recommended outside a clinical trial where there is little or negative evidence of ICI sensitivity without compelling biological rationale, a clearly superior evidence-based alternative exists, or the risk of serious toxicity is judged to outweigh the plausible benefit (Table 2).

The framework is intentionally not an additive numerical score, because these factors are neither independent nor equivalently weighted across tumour types. Strong clinical or biological evidence may outweigh uncertainty in other domains, whereas major toxicity concerns or the availability of a clearly superior standard therapy may override otherwise favourable features. This approach therefore provides structured guidance while preserving the clinical judgement required when evidence is incomplete.

Australia has therefore created a national implementation experiment. The best-case scenario is that broad reimbursement improves equity for patients with rare and uncommon cancers, while real-world evidence clarifies where benefit is greatest. The worst-case scenario is more variable care, avoidable toxicity, and treatment of biologically unlikely responders because the system funded the drug before it funded the selection pathway.

The policy response and international learnings from this approach are significant. Early lessons from Australia's approach should be to build the infrastructure around access. This includes public funding for appropriate molecular testing, structured multidisciplinary review, including rare-cancer and molecular tumour boards where available, clinician-facing guidance, toxicity pathways, and prospective registries that capture tumour type, biomarker status, rationale for treatment, dose and schedule, toxicity, response, duration of benefit, and survival outcomes.

## From affordability to appropriate use

For nearly a decade, cost has been the primary barrier to broad immunotherapy reimbursement, particularly in health systems with constrained resources. In the United States, annual treatment for PD-1/L1 inhibitors commonly exceed US$100,000 per patient,^48^ and multiple cost-effectiveness analyses have questioned whether broad population-level use is sustainable at originator prices.^49^^,^^50^ However, the economic landscape is evolving as competition, biosimilar development, alternative procurement models, and broader reimbursement pathways emerge. These changes are unlikely to eliminate affordability concerns uniformly across jurisdictions, but they may progressively reduce price as the sole determinant of access in some settings.^51^

As access broadens, the parallel question of appropriate use becomes increasingly important: which patients are sufficiently likely to benefit to justify treatment-related toxicity, resource use, and opportunity cost? Severe immune-related adverse events occur in 15–20% of patients receiving checkpoint inhibitors, with fatal outcomes in 0.3–1.2% of cases.^52^^,^^53^ In this setting, reimbursement policy should not be considered independently of patient selection.

As financial barriers evolve, “clinical judgment” becomes an increasingly important determinant of responsible access. The clinician's responsibility shifts from defending cost-benefit ratios to making individualized decisions about immunotherapy candidacy based on rigorous patient selection criteria. The framework proposed in Table 2 therefore addresses not only whether immunotherapy can be accessed, but whether the available clinical evidence, biological rationale, competing treatment options, and patient-specific benefit–risk support its use in an individual patient. Such tools operationalise the translation from genomic and immunologic data into actionable clinical guidance, helping practitioners identify patients most likely to benefit while protecting those at highest risk of severe toxicity.

## Broader lessons for emerging tumour-agnostic therapies

The principles emerging from tumour-agnostic immunotherapy are relevant to newer pan-tumour therapeutic platforms, including antibody-drug conjugates (ADCs), bispecific antibodies, and cellular therapies. Shared target expression among tumour types does not necessarily define a conserved therapeutic vulnerability.^4^^,^^5^ For ADCs for example, target density, internalization kinetics, spatial heterogeneity, payload sensitivity, tumour lineage, and resistance mechanisms may all influence efficacy despite expression of the same antigen.^54^^,^^55^ Similar context dependence is likely to apply to other target-directed immune-oncology modalities. The framework proposed for tumour-agnostic immunotherapy therefore has broader relevance: pan-tumour development should integrate the strength of clinical evidence, biological validity of the therapeutic target, assay characteristics and tumour-lineage context, and patient-specific benefit–risk rather than relying on target positivity alone. Where evidence is initially derived from small basket cohorts or uncommon cancers, prospective outcome capture and iterative refinement of selection biomarkers should accompany expansion of access. In this way, lessons from tumour-agnostic immunotherapy may help emerging platforms avoid translating a measurable pan-cancer feature prematurely into a universal predictor of treatment benefit.

## Conclusions and future directions

Tumour-agnostic immunotherapy has not made tumour type obsolete. It has shown that, in selected settings, biology can be more clinically informative than anatomy. MSI-H/dMMR remains the clearest example because it captures a conserved immune-sensitive state. TMB, rare cancer immunotherapy, and emerging pan-tumour drug classes illustrate the other side of the same lesson: a biomarker that is measurable across cancers is not automatically meaningful across cancers. While histology-based drug development will continue to dominate common cancers (lung, breast, prostate, colorectal), biomarker-driven therapies address a critical gap, extending treatment options to rare cancer patients who would otherwise have limited options.

The next phase should be judged not only by whether more patients can receive treatment, but by whether health systems can identify likely responders, support toxicity, avoid low-value care, and learn from every patient treated. Biological rationale must align with biomarker precision; biomarker precision must align with trial design; and finally access must align with testing infrastructure, clinician support, toxicity pathways, and real-world data systems. Australia's broad reimbursement model for immunotherapy-sensitive cancers offers a valuable test case for improving equity in rare and uncommon cancer treatment. However, its success depends on four critical prerequisites: molecular testing to identify patients with actionable biomarkers, expert clinical decision support to ensure appropriate patient selection and treatment planning, prospective registries to track outcomes and safety in real-world settings, and mechanisms for reassessment to adjust coverage and recommendations based on emerging evidence. Without these safeguards, the model risks both waste and harm.

## Contributors

JL conceived the Review and developed the overall scope and structure. JL, NC, and JB designed and led the search strategy and selection criteria. All authors contributed to drafting the manuscript, critically reviewed and revised it for important intellectual content, approved the final version for submission, and accept responsibility for the decision to submit for publication.

## Declaration of interests

JLiu: Research Grants: MSD, Merck, Guardant Health; Honoraria: Taiho Oncology, MSD, Merck, Dr Reddy's; Consulting or Advisory Role: Starpharma (Inst), Greywolf Therapeutics, Daiichi Sankyo, Kivu Biosciences; Participation on DSMB: Starpharma, Relay Therapeutics, IDEAYA Biosciences, Innovent Biologics, Greywolf Therapeutics, Nested Therapeutics, Carina Biotech, ImmVirX, MediLink Therapeutics, Kivu Biosciences, BeOne. Research Funding: Starpharma (Inst), Relay Therapeutics (Inst), ALX Oncology (Inst), IDEAYA Biosciences (Inst), Innovent Biologics (Inst), Greywolf Therapeutics (Inst), MSD (Inst), Regeneron (Inst), Bristol Myers Squibb (Inst), AbbVie (Inst), AVEO (Inst), Bayer (Inst), Merus (Inst), Nested Therapeutics (Inst), Bristol Myers Squibb (Inst), Carina Biotech (Inst), ImmVirX (Inst), AVEO (Inst), Daiichi Sankyo/UCB Japan (Inst), MediLink Therapeutics (Inst), Covus Therapeutics (Inst), OncoNano Inc (Inst), Kivu Biosciences, Guardant Health (Inst), BeOne (Inst); Travel, Accommodations, Expenses: Starpharma, Innovent Biologics.

NC: Travel, Accommodations, Expenses: Pfizer, Novartis, MSD.

JB: Honoraria: AstraZeneca, Daiichi Sankyo/AstraZeneca, Travel, Accommodations, Expenses: AstraZeneca/Daiichi Sankyo.

JLyn: Consulting or advisory role: Precision for Medicine Global CRO, BAYER, Janssen, MSD (Inst); Speaker Fees: BAYER, AstraZeneca, Janssen; BMS (Inst), Ipsen (Inst).

VS: Consulting or Advisory Role: Loxo/Lilly, Relay Therapeutics (Inst), Pfizer (Inst), Roche (Inst), Bayer (Inst), Incyte (Inst), Novartis (Inst), Pheon Therapeutics (Inst), AbbVie (Inst), Illumina, AADi, Foundation Medicine; Research Funding: Novartis (Inst), GlaxoSmithKline (Inst), NanoCarrier (Inst), Northwest Biotherapeutics (Inst), Genentech/Roche (Inst), Berg Pharma (Inst), Bayer (Inst), Incyte (Inst), Fujifilm (Inst), PharmaMar (Inst), D3 Oncology Solutions (Inst), Pfizer (Inst), Amgen (Inst), AbbVie (Inst), Multivir (Inst), Blueprint Medicines (Inst), LOXO (Inst), Vegenics (Inst), Takeda (Inst), Alfasigma (Inst), Agensys (Inst), Idera (Inst), Boston Biomedical (Inst), Inhibrx (Inst), Exelixis (Inst), Amgen (Inst), Turning Point Therapeutics (Inst), Relay Therapeutics (Inst); Other Relationship: Medscape, Clinical Care Options.
